# Comparing Complication Rates, Costs, and Length of Stay between Unicompartmental and Total Knee Arthroplasty: Insights from a Big Data Analysis Using the National Inpatient Sample Dataset

**DOI:** 10.3390/jcm13133888

**Published:** 2024-07-02

**Authors:** David Maman, Assil Mahamid, Yaniv Yonai, Yaron Berkovich

**Affiliations:** 1 Rappaport Faculty of Medicine, Technion University Hospital (Israel Institute of Technology), Haifa 3200003, Israel; yanivyonai@gmail.com (Y.Y.); yaron.berkovich@gmail.com (Y.B.); 2 Department of Orthopedics, Carmel Medical Center, Haifa 3436212, Israel; 3 Department of Orthopedics, Hillel Yaffe Medical Center, Hadera 3200003, Israel; assil10@gmail.com

**Keywords:** UKA, unicompartmental knee arthroplasty, TKA, total knee arthroplasty, NIS

## Abstract

**Background:** Unicompartmental knee arthroplasty (UKA) is increasingly used for knee osteoarthritis due to faster recovery, better range of motion, and lower costs compared to total knee arthroplasty (TKA). While TKA may offer longer-lasting results with lower revision rates, this study compares the relative benefits and limitations of UKA and TKA using the National Inpatient Sample (NIS) database. **Methods:** This retrospective analysis examined outcomes of elective UKA and TKA procedures from 2016 to 2019, identifying 2,606,925 patients via ICD-10 codes. Propensity score matching based on demographics, hospital characteristics, and comorbidities resulted in a balanced cohort of 136,890 patients. The present study compared in-hospital mortality, length of stay, postoperative complications, and hospitalization costs. **Results:** The results showed that UKA procedures increased significantly over the study period. Patients undergoing UKA were generally younger with fewer comorbidities. After matching, both groups had low in-hospital mortality (0.015%). UKA patients had shorter hospital stays (1.53 vs. 2.47 days) and lower costs (USD 55,976 vs. USD 61,513) compared to TKA patients. UKA patients had slightly higher rates of intraoperative fracture and pulmonary edema, while TKA patients had higher risks of blood transfusion, anemia, coronary artery disease, pulmonary embolism, pneumonia, and acute kidney injury. **Conclusions:** UKA appears to be a less-invasive, cost-effective option for younger patients with localized knee osteoarthritis.

## 1. Introduction

Unicompartmental knee arthroplasty (UKA) and total knee arthroplasty (TKA) represent crucial surgical options for managing knee osteoarthritis, each with distinct implications for patient outcomes. As surgical paradigms evolve and indications for UKA expand, its utilization is increasing [1,2], with studies suggesting that up to 20% of osteoarthritis patients could benefit from this targeted approach [3]. UKA offers the advantage of preserving the healthy knee compartment, which can lead to shorter recovery times and superior functional outcomes, particularly regarding range of motion and pain reduction during recovery [4,5]. In contrast, while TKA entails a longer initial recovery, it often provides significant long-term relief and enhanced joint function [5,6].

UKA has several economic advantages. Shorter hospital stays, reduced blood loss, and consequently, fewer blood transfusions translate to decreased overall healthcare costs [7,8,9,10]. These benefits make UKA an increasingly attractive option for well-selected patients. However, the decision between UKA and TKA is highly individualized. Several patient factors must be meticulously considered, including the severity and location of the arthritis, age and activity level, and overall health status. Additionally, implant longevity and the potential need for revision surgery, which is typically lower with TKA, are crucial considerations [11].

Recent studies highlight that patients undergoing UKA experience reduced postoperative pain, return to work more swiftly, and have an enhanced range of motion compared with those treated with TKA [12,13,14]. While some studies question the superiority of UKA over TKA, they consistently affirm that UKA achieves at least equivalent functional outcomes, thereby supporting its use as an effective alternative in appropriate clinical scenarios [15,16].

In the past three years, there has been an increasing body of research supporting the advantages of UKA in terms of shorter hospital stays and reduced costs. Studies by Smith et al. [17] and Johnson et al. [18] have demonstrated that UKA not only reduces immediate postoperative complications, but also results in lower overall healthcare expenditure compared to TKA. Furthermore, recent advancements in surgical techniques and postoperative care have further enhanced the outcomes of UKA, making it a viable option for a broader range of patients [17,18].

Our study, utilizing data from the National Inpatient Sample (NIS) involving 68,445 UKA patients and 2,538,480 TKA patients, seeks to compare hospitalization characteristics, complications, and costs associated with these procedures. This comprehensive analysis aims to enhance our understanding of the practical implications, benefits, and limitations of UKA relative to TKA, providing valuable insights that could influence future advancements in patient-centered care and healthcare resource allocation.

## 2. Methods

### 2.1. Data Source and Study Population

This retrospective analysis utilized data from the National Inpatient Sample (NIS), a large administrative database that captures inpatient stays across the United States. The NIS database is maintained by the Healthcare Cost and Utilization Project (HCUP) and provides a representative sample of hospital discharges. We included patients who underwent UKA or TKA, identified using specific ICD-10 procedure codes.The study period spanned from 1 January 2016 to 31 December 2019, which are the latest available data within the NIS system at the time of this study.

### 2.2. Inclusion and Exclusion Criteria

Patients undergoing elective UKA or TKA during the study period were included. Elective procedures were identified based on admission type. We excluded patients with non-elective admissions (e.g., emergency admissions), prior knee surgery, or revision surgeries to maintain data homogeneity. This yielded a cohort of 2,606,925 patients: 68,445 undergoing UKA and 2,538,480 undergoing TKA.

### 2.3. Propensity Score Matching

To minimize confounding factors, propensity score matching was performed using MATLAB 2024. We matched patients undergoing UKA to those undergoing TKA based on several characteristics, including age, sex, race, hospital size, patient location (urban/rural), median household income quartile by ZIP Code, hospital region, total discharges within the NIS dataset, comorbidities (such as hypertension, diabetes, and obesity), and payer type (Medicare, Medicaid, private insurance, self-pay, etc.). This process resulted in a balanced dataset of 136,890 patients (68,445 UKA and 68,445 TKA).

### 2.4. Outcome Measures

Following propensity score matching, we compared the following outcomes between the UKA and TKA groups: in-hospital mortality, length of stay, postoperative complications, and overall hospitalization costs.

### 2.5. Statistical Analysis

Statistical analyses were conducted using SPSS 26. We used chi-square tests to compare categorical variables and independent samples; we used *t*-tests to compare continuous variables. Risk ratios with 95% confidence intervals were calculated for each complication. A significance level of *p* < 0.05 was used for all analyses.

### 2.6. Ethical Considerations

This study received exempt status from the institutional review board due to the de-identified nature of the NIS dataset.

## 3. Results

**Figure 1:**
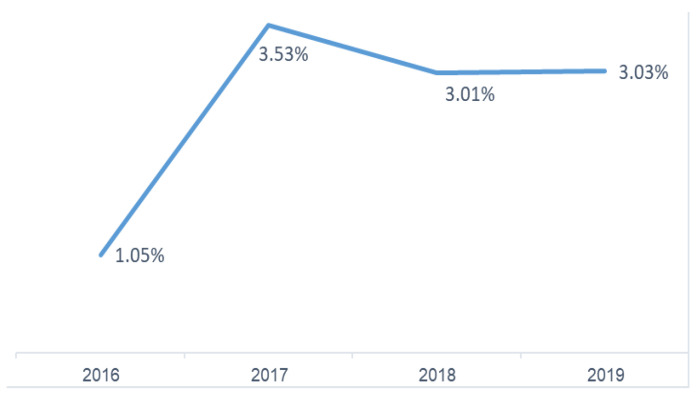
Percentage of UKA procedures among all knee arthroplasty procedures (2016–2019).

Our analysis of the Nationwide Inpatient Sample from 2016 to 2019 investigated UKA relative to TKA procedures. Examining the proportion of UKA procedures among all knee arthroplasties, we observed a statistically significant increase (*p* < 0.0001) in the prevalence of UKAs over the study period, as shown in Figure 1. This trend manifested as a sharp rise in the percentage of UKA procedures from 1.05% in 2016 to 3.53% in 2017, while the utilization of UKA from 2017 to 2019 plateaued.

Primary osteoarthritis is the leading cause for both UKA and TKA procedures, as shown in Table 1. It accounts for nearly all surgeries: 97.35% for UKA and 97.70% for TKA. Post-traumatic arthritis is the second most common etiology, affecting a small percentage of patients undergoing both procedures (1.13% for UKA and 1.46% for TKA). Rheumatoid arthritis, osteonecrosis, leg deformity, malignant neoplasm, and other unspecified etiologies are less frequent causes for both UKA and TKA.

To understand potential differences between UKA and TKA patients, we analyzed their demographic and clinical characteristics before applying propensity score matching. This analysis included all available data.

Our analysis revealed notable differences between the two groups. Patients undergoing UKA tended to be younger than those undergoing TKA. Additionally, the prevalence of comorbidities differed between the groups.

Table 2 provides a detailed breakdown of various parameters for UKA and TKA patients before propensity score matching. It highlights key differences in age, sex, payer information, and the prevalence of specific comorbidities.

In order to overcome potential selection bias and baseline differences, a propensity score-matched analysis was performed. As discussed in Section 2, selection bias can arise when comparing outcomes between UKA and TKA procedures. To address this and ensure a fair comparison, we employed this statistical technique as it balances baseline characteristics between the two groups. This approach ensures that any observed differences in outcomes can be more confidently attributed to the type of surgery itself, rather than pre-existing variations between the patient populations undergoing UKA and TKA.

After propensity score matching, the two groups were observed to be statistically equivalent across all of the parameters presented in Table 3. This indicates that the propensity score matching method successfully balanced the baseline characteristics between patients undergoing UKA and TKA, ensuring that any observed differences in outcomes could be attributed to the type of surgery rather than underlying patient demographics or comorbidities.

In our analysis comparing hospitalization outcomes between UKA and TKA in propensity score-matched cohorts, several key findings emerged as shown un Table 4. Firstly, the incidence of mortality during hospitalization was found to be low in both UKA and TKA groups, with rates of 0.015% for each. However, disparities were observed in the length of hospital stay and total charges incurred. Patients undergoing UKA had a significantly shorter mean length of stay compared to those undergoing TKA (1.53 days vs. 2.47 days, respectively; *p* < 0.0001). Additionally, TKA was associated with higher mean total charges compared to UKA by USD 5537.

In our investigation comparing postoperative complications between UKA and TKA in propensity score-matched cohorts, several important findings emerged. As shown in Table 5, our analysis revealed that UKA did not demonstrate superiority over TKA in terms of some postoperative complications. UKA exhibited higher rates of intraoperative fracture and pulmonary edema compared to TKA, with statistically significant differences observed in both instances (*p* = 0.007 and *p* < 0.0001, respectively). While the incidence of venous thromboembolism was slightly lower in UKA compared to TKA, this difference was not statistically significant (*p* = 0.06). Similarly, there was no significant difference in the incidence of heart failure between the two procedures (*p* = 0.44).

**Figure 2:**
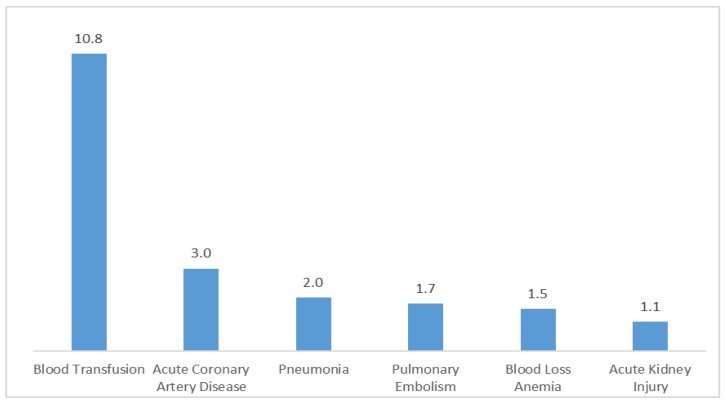
Increased risk of postoperative complications with TKA compared to UKA in propensity score-matched cohorts.

In Figure 2, the risk estimates illustrate the increased likelihood of experiencing various postoperative complications when opting for TKA over UKA. Risk signifies the elevated probability or chance of encountering a specific complication following TKA compared to UKA. The risk estimates quantify this increased likelihood, delineating the relative rise in risk associated with TKA for each complication as follows:

Blood transfusion: the risk of requiring a blood transfusion following TKA is substantially elevated by a factor of 10.812 compared to UKA (95% CI: 8.353–13.996, *p* < 0.0001). Blood loss anemia: TKA is associated with a 54.7% increase in the risk of developing blood loss anemia relative to UKA (risk estimate: 1.547, 95% CI: 1.509–1.585, *p* < 0.0001). Acute coronary artery disease: the likelihood of experiencing acute coronary artery disease postoperatively is notably higher with TKA, presenting a 200.5% increase in risk compared to UKA (risk estimate: 3.005, 95% CI: 2.385–3.787, *p* < 0.0001).

Pulmonary embolism: opting for TKA over UKA entails a 72.3% rise in the risk of pulmonary embolism (risk estimate: 1.723, 95% CI: 1.347–2.204, *p* < 0.0001). Pneumonia: TKA is associated with a 95.1% higher risk of developing pneumonia postoperatively compared to UKA (risk estimate: 1.951, 95% CI: 1.536–2.478, *p* < 0.0001). Acute kidney injury: TKA presents a 7.2% higher risk compared to UKA (risk estimate: 1.072, 95% CI: 1.011–1.137, *p* = 0.017). These risk estimates elucidate the comparative increase in the likelihood of experiencing these complications following TKA relative to UKA.

## 4. Discussion

Our investigation, utilizing the Nationwide Inpatient Sample from 2016 to 2019, highlighted a significant increase in the utilization of UKA compared to TKA, particularly noting a surge from 1.05% in 2016 to 3.53% in 2017. This significant increase in UKA procedures aligns with recent trends reported in the literature [1,2]. While TKA remains the predominant surgery for severe knee conditions, especially in older adults, UKA has gained favor due to its less invasive nature and faster recovery times. Yet many surgeons still tend to choose TKA over UKA due to its proven efficacy, lower revision rates, and higher patient satisfaction [17]. Unsurprisingly, the primary etiology for both surgical interventions was predominantly primary osteoarthritis, accounting for over 97% of cases, with other causes like post-traumatic and rheumatoid arthritis being far less common.

### 4.1. Costs

Our comprehensive analysis of hospitalization outcomes in matched cohorts undergoing UKA and TKA revealed a significantly shorter LOS in the UKA group. This finding aligns with previous studies demonstrating similar reductions in LOS [18,19]. Moreover, some studies have even explored the comparison of lateral compartment arthroplasty to TKA [7]. From a financial perspective, the shorter LOS associated with UKA translates to substantial cost savings for the hospital. Our analysis suggests a potential annual saving of 1817.4 inpatient ward bed days, corresponding to an estimated cost reduction of USD 2,124,540.60 and up to USD 2397.28 per patient [20]. Furthermore, our data demonstrate a statistically significant difference in total charges, with the TKA group incurring an additional USD 5537 compared to the UKA group. Supporting this finding, a prior study reported not only lower direct hospital costs for UKA, but also shorter anesthesia and operative times, further contributing to reduced overall costs [9].

### 4.2. Complications

In our study, UKA was associated with slightly higher rates of intraoperative fractures and pulmonary edema compared to TKA. These complications, although relatively infrequent, can have significant clinical implications. Intraoperative fractures may necessitate additional surgical intervention, potentially delaying recovery and increasing healthcare costs [18]. Pulmonary edema, although rare, requires prompt management to prevent serious outcomes such as respiratory failure.

TKA was linked to higher rates of several serious complications, including blood transfusion, blood loss anemia, acute coronary artery disease, pulmonary embolism, pneumonia, and acute kidney injury. These findings highlight the necessity for careful patient selection and surgical planning to mitigate risks and optimize outcomes for both procedures.

### 4.3. Pros of UKA

UKA offers several significant advantages over TKA. One of the primary benefits is the preservation of healthy knee compartments, which contributes to a quicker recovery and less postoperative pain [8,9,11]. Patients undergoing UKA typically experience a shorter hospital stay and lower overall healthcare costs due to reduced blood loss and fewer complications, as shown in our study and in previous studies [8,20,21,22]. Additionally, UKA patients often achieve better postoperative range of motion and a higher level of activity at the time of discharge compared to those undergoing TKA. This improved functional outcome is particularly beneficial for younger, more active patients. Furthermore, UKA is associated with a lower incidence of some serious complications, such as blood transfusion and acute coronary artery disease, making it an attractive option for well-selected patients.

### 4.4. Cons of UKA

UKA offers several advantages, but its broader adoption is tempered by concerns regarding potentially higher revision rates. Data from the German Arthroplasty Registry (EPRD) suggest an increased risk of early failure (after 12 months) with UKA compared to TKA, with this risk doubling by the four-year mark [23]. Notably, some of this disparity has been attributed to the procedure being performed in low-volume hospitals [24,25]. Additionally, a higher body mass index (BMI) has been correlated with a propensity for revision surgery in UKA patients [26].

### 4.5. Limitations and Strengths

This study leverages a large, nationally representative dataset to compare real-world outcomes of UKA and TKA. Propensity score matching mitigates confounding factors, strengthening the comparison. However, the use of administrative data from the NIS limits the analysis to in-hospital outcomes and lacks long-term patient perspectives. Additionally, the retrospective nature of the data and potential coding errors might influence the results [27]. Future research should incorporate long-term follow-up and patient-reported measures for a more holistic picture.

Despite these limitations, the present study’s large sample size and comprehensive analysis offer valuable insights into patient demographics, clinical characteristics, hospitalization details, and cost implications of UKA vs. TKA. These findings highlight shorter hospital stays and lower costs for UKA, alongside differing complication profiles. This valuable contribution fuels the discussion around optimal surgical approaches for knee osteoarthritis, paving the way for future research that ultimately improves patient care and resource allocation in knee arthroplasty.

## 5. Conclusions

This study shows the importance of understanding the trade-offs between UKA and TKA for optimal surgical decision-making. By highlighting UKA’s advantages in terms of shorter hospital stays, reduced costs, and a lower incidence of certain complications, this study empowers healthcare providers and patients in knee arthroplasty decisions. However, the present study’s limitations include reliance on administrative data, a lack of long-term follow-up, and potential coding errors. Future research should focus on long-term outcomes and patient-reported measures to provide a more comprehensive understanding of UKA and TKA’s impact on quality of life, with ongoing advancements in surgical techniques and care protocols expected to improve patient outcomes and satisfaction.

## Figures and Tables

**Table 1:** Distribution of etiologies for UKA and TKA.

Etiologies	UKA (%)	TKA (%)	Significance
Primary osteoarthritis	97.35	97.70	*p* < 0.0001
Post-traumatic arthritis	1.13	1.46	
Rheumatoid arthritis	0	0.22	
Osteonecrosis	0.52	0.06	
Leg deformity	0	0.06	
Malignant neoplasm	0.02	0.02	
Other/unspecified	0.98	0.48	

**Table 2:** Patient characteristics before propensity score matching.

Parameter	UKA	TKA	Significance
Total Surgeries (%)	68,445	2,538,480	-
Average Age (y)	63.23 (Std d.10.6)	66.76 (Std d.9.416)	*p* < 0.0001
Female (%)	50.5	61.7	*p* < 0.0001
Payer—Medicare (%)	40.8	57.3	*p* < 0.0001
Payer—Medicaid (%)	5.1	4.3	
Payer—Private (%)	48.5	34.9	
Payer—Other (including self-pay) (%)	5.6	3.5	
Hypertension Diagnosis (%)	52.2	59.5	*p* < 0.0001
Dyslipidemia Diagnosis (%)	42.4	46.6	*p* < 0.0001
Sleep Apnea Diagnosis (%)	12.4	13.2	*p* < 0.0001
Chronic Anemia (%)	2.8	5.9	*p* < 0.0001
Alcohol Abuse (%)	0.8	0.9	*p* = 0.103
Osteoporosis (%)	2.4	4	*p* < 0.0001
Parkinson’s Disease (%)	0.5	0.6	*p* < 0.0001
Type 2 Diabetes (%)	16.5	21.5	*p* < 0.0001
Renal Disease (%)	4.8	7	*p* < 0.0001
CHF (%)	0.7	1.3	*p* < 0.0001
Chronic Lung Disease (%)	4.4	6.0	*p* < 0.0001
Obesity (%)	25.5	31.1	*p* < 0.0001

**Table 3:** Patient characteristics after propensity score matching.

Parameter	UKA	TKA	Significance
Total Surgeries (%)	68,445	68,445	-
Average Age (y)	63.23 (Std d.10.6)	63.28 (Std d.10.5)	*p* = 0.75
Female (%)	50.5	50.9	*p* = 0.10
Payer—Medicare (%)	40.8	41.1	*p* = 0.23
Payer—Medicaid (%)	5.1	5	
Payer—Private (%)	48.5	48.4	
Payer—Other (including self-pay) (%)	5.6	5.5	
Hypertension Diagnosis (%)	52.2	52.4	*p* = 0.35
Dyslipidemia Diagnosis (%)	42.4	42.6	*p* = 0.43
Sleep Apnea Diagnosis (%)	12.4	12.1	*p* = 0.08
Chronic Anemia (%)	2.8	2.7	*p* = 0.51
Alcohol Abuse (%)	0.8	0.8	*p* = 0.77
Osteoporosis (%)	2.4	2.4	*p* = 0.861
Parkinson’s Disease (%)	0.5	0.5	*p* = 0.69
Type 2 Diabetes (%)	16.5	16.3	*p* = 0.309
Renal Disease (%)	4.8	4.7	*p* = 0.31
CHF (%)	0.7	0.6	*p* = 0.06
Chronic Lung Disease (%)	4.4	4.3	*p* = 0.236
Obesity (%)	25.5	25.2	*p* = 0.29

**Table 4:** Comparison of hospitalization outcomes in propensity score-matched cohorts.

Parameter	UKA	TKA	Significance
Died during hospitalization	0.015%	0.015%	*p* = 1
Length of stay mean in days	1.53 (Std d.1.4)	2.47 (Std d.1.5)	*p* < 0.0001
Mean total charges in USD	55,976 (Std d.34,156)	61,513 (Std d.38,569)	*p* < 0.0001

**Table 5:** Comparison of postoperative complications, where UKA matches or exceeds TKA in propensity score-matched cohorts.

Parameter	UKA (%)	TKA (%)	Significance
Intraoperative fracture	0.23	0.17	*p* = 0.007
Pulmonary edema	0.06	0.02	*p* < 0.0001
Venous thromboembolism	0.16	0.20	*p* = 0.06
Heart failure	0.10	0.04	*p* = 0.44

## Data Availability

The data presented in this study are available on request from the corresponding author. The data are not publicly available due to privacy.

## Abbreviations

HCUP	Healthcare Cost and Utilization Project
ICD-10	International Classification of Diseases, 10th Revision
LOS	Length of stay
NIS	Nationwide Inpatient Sample
SPSS	Statistical Package for the Social Sciences
TKA	Total hip arthroplasty
UKA	Unicompartmental knee arthroplasty
